# Who Regulates Whom? An Overview of RNA Granules and Viral Infections

**DOI:** 10.3390/v8070180

**Published:** 2016-06-28

**Authors:** Natalia Poblete-Durán, Yara Prades-Pérez, Jorge Vera-Otarola, Ricardo Soto-Rifo, Fernando Valiente-Echeverría

**Affiliations:** 1 Molecular and Cellular Virology Laboratory, Virology Program, Institute of Biomedical Sciences, Faculty of Medicine, Universidad de Chile, Independencia 1027, Santiago, 8389100, Chile; natalia.poblete.duran@gmail.com (N.P.-D.); ypradesp@ug.uchile.cl (Y.P.-P.); rsotorifo@med.uchile.cl (R.S.-R.); 2 Laboratorio de Virología Molecular, Instituto Milenio de Inmunología e Inmunoterapia, Centro de Investigaciones Médicas, Departamento de Enfermedades Infecciosas e Inmunología Pediátrica, Escuela de Medicina, Pontificia Universidad Católica de Chile, Marcoleta 391, Santiago 8330024, Chile; javochile@gmail.com

**Keywords:** RNA granules, stress granules, P-bodies, anti-viral host immune response, translation control

## Abstract

After viral infection, host cells respond by mounting an anti-viral stress response in order to create a hostile atmosphere for viral replication, leading to the shut-off of mRNA translation (protein synthesis) and the assembly of RNA granules. Two of these RNA granules have been well characterized in yeast and mammalian cells, stress granules (SGs), which are translationally silent sites of RNA triage and processing bodies (PBs), which are involved in mRNA degradation. This review discusses the role of these RNA granules in the evasion of anti-viral stress responses through virus-induced remodeling of cellular ribonucleoproteins (RNPs).

## 1. Introduction

The control of the translation and turnover of mRNAs plays fundamental roles in the regulation of gene expression. Eukaryotic cells have developed different mechanisms to control these processes in order to respond to environmental stress, such as heat shock, UV irradiation, hypoxia, endoplasmic reticulum (ER) stress and viral infection. Among those mechanisms is the assembly of non-membrane-delimited bodies, called RNA granules, that contain RNA-binding proteins (RBPs) and translationally silenced mRNAs [1,2].

The classification of RNA granules is based on a number of factors, including their subcellular localization (nuclear, cytoplasmic, axonal, etc.), the presence of specific markers, the cell type where they are assembled (germ cells, neurons), response to stimuli (stress, viral infection), dynamic behavior and proposed functions (sites of mRNA storage/decay, stress response, etc.) [3]. The nucleus contains the nucleolus, nuclear speckles, nuclear stress bodies, the transcription factory, Cajal bodies, the Gemini of Cajal bodies, the histone locus body, paraspeckles and PML bodies, all of which have specific regulatory functions (reviewed in [4,5]). The cytoplasm harbors the polar and germinal granules, stress granules (SGs), processing bodies (P-bodies or PBs) and neuronal granules [6,7]. Many RNA viruses have evolved mechanisms to modulate the assembly of different RNA granules. In this review, we will summarize the regulation of SGs and PBs by different virus families.

## 2. RNA Granules: Stress Granules (SBs) and Processing Bodies (PBs)

SGs and PBs are associated with mRNA storage and decay, respectively [8]. Importantly, increasing evidence suggests that abnormal SG formation may promote several neurodegenerative disorders, including amyotrophic lateral sclerosis (ALS), frontotemporal lobar degeneration (FTLD) and spinal muscular atrophy (SMA) [9]. The formation of SGs in response to chemotherapeutic treatments has also been associated with cancer cell survival [3]. SGs are specifically induced upon cellular stress [10], triggering global translational silencing typically through the phosphorylation of the translation initiation factor eIF2α. Four eIF2α kinases sense environmental stress: HRI (heme-regulated eIF2α kinase) is activated in heme deprivation and oxidative stress [11]; PKR, (Protein Kinase RNA-dependent) is a double-stranded RNA (dsRNA)-dependent protein kinase activated by viral infection [12]; PERK/PEK (PKR-like ER kinase) is activated during hypoxia and in response to misfolded proteins in the ER [13]; and GCN2 (general control non-derepressible-2) is activated during amino acid depravation and UV irradiation [14]. The assembly of SGs can also occur independently of eIF2α phosphorylation, upon treatment with hippuristanol or pateamine A, drugs that inhibit the helicase activity of eIF4A [15,16]; oligomycin, FCCP or 2-deoxy-D-glucose, metabolic inhibitors that deplete cellular adenosine triphosphate [17]; hydrogen peroxide, a known inducer of reactive oxygen species [18]; as well as overexpression of the SG markers TIA1/TIAR [19] or G3BP-1 [20]. Recently, Kedersha and colleagues reported that SG assembly is finely regulated by the binding of G3BPs to Caprin1 or USP10 [21]. By contrast, PBs are constitutively present and respond to stimuli that affect mRNA translation and decay [7]. Three proteins are known to play a critical role in PB assembly, Edc3, Pat1 and Lsm4 [22,23], due to their Q/N-rich domains that have the potential to form aggregates [24]. PBs can release mRNA to allow their translation [25,26,27]. Additionally, emetine and cycloheximide, drugs that stabilize polysomes, dissolve both SGs and PBs, whereas puromycin, a drug that disrupts polysomes, promotes their assembly [27].

### 2.1. SG Components

SGs are composed of target mRNAs bound to translation initiation factors (eIF4G, eIF3, PABPC1, eIF2α-P, eIF5A) to allow the rapid restoration of translation once the stress is gone [19,28]. Many other proteins are involved in SG formation, such as mRNA binding proteins that provide translational control or mRNA stability (TIA-1, TIAR, HuR/ELAVL1, FXR1, Pum1) [6,29,30,31], proteins related to mRNA metabolism (G3BP1, G3BP2, p54/rck/DDX6, PMR1, SMN, Staufen1, DHX36, Caprin1, ZBP1, HDAC6, ADAR) [20,32,33,34,35,36] and signaling proteins (mTOR, RACK1, TRAF2) [37]. Moreover, interferon-stimulated gene (ISG) products, such as, PKR, ADAR1, RNA-sensing RIG-I-like receptors (RIG-I, MDA5, LGP2), RNase L and OAS, have been shown to colocalize with SGs following viral infections [38,39]. 

### 2.2. PB Components

PBs contain components of the mRNA decapping machinery (Dcp1/2, Lsm1-7, Edc3proteins) [40,41], scaffolding proteins (Ge-1/Hedls) [41], deadenylation factors (Ccr1, Caf1, Not1) [42], nonsense-mediated decay (NMD) proteins (SMG5-6-7, UPF1) [40,43] and translation control factors (CPEB, eIF4E-T) [32,44]. Most recently, Patel and colleagues showed that GW-bodies, which also are ribonucleoproteins (RNPs) involved in mRNA decay, are distinct from P-bodies. GW-bodies are unique in that they contain GW182, a large scaffolding protein containing an N-terminal domain composed of GW/WG motifs. The N-terminal GW/WG motif-bearing domain has been shown to bind to AGO2, while the C-terminus interacts with the CCR4-NOT deadenylation complex [45]. Moreover, GW-bodies have a different spatial-temporal regulation because they play a major functional role in miRNA-mediated gene silencing rather than degradation [45]. As a result of virus infection, ISGs can also be found in PBs [46]. Given that SGs and PBs are highly dynamic, many proteins have been described as being part of both RNA granules (APOBEC3G, Ago2, BRF1, DDX3, FAST, TTP, etc.) [29,47,48,49], suggesting that mRNAs can move between both mRNPs, thus regulating RNA homeostasis. A comprehensive list of SG, PB and SG/PB components is provided in Table 1.

## 3. Viral Infection and Stress Granules

Several viruses modulate the assembly of SGs to promote viral replication and suppress the cellular stress response. The discussion below was performed upon the interaction between virus families and SGs (Table 2).

### 3.1. Double-Stranded DNA (dsDNA) Viruses

Members of the family *Herpesviridae* and *Poxviridae* are enveloped double-stranded DNA (dsDNA) viruses. Herpes simplex virus type 1 (HSV-1) shuts off host protein synthesis by impairing the activation of eIF2 kinases through the virion host shutoff (vhs) protein [129], Us11 [130], ICP34.5 [131] and glycoprotein B (gB) [132]. During HSV-1 infection, the SG components TIA-1, TIAR and TTP are upregulated, but do not form SG, however, infection with vhs-defective HSV-1 triggers SG assembly [133,134], relying on PKR activity in the absence of vhs [135]. Recently, Finnen and colleagues have shown that infection with HSV type 2 (HSV-2) impairs arsenite-mediated SG assembly, while the SG induced by treatment with pateamine A are not affected [136]. The blockage in arsenite-induced SG assembly is dependent on vhs, as cells infected with a vhs defective HSV-2 mutant form SGs late during infection [137]. Human cytomegalovirus (HCMV) infection suppresses the assembly of SGs in cells treated with the ER stressor thapsigargin [138], while simultaneously inducing an unfolded protein response (UPR) and activating PERK, but limiting eIF2α phosphorylation to maintain viral RNA translation [139].

Vaccinia virus (VV), a member of *Poxviridae* family, replicates within the cytoplasm in large foci called DNA factories that co-opt SG proteins, such as G3BP1, Caprin1, eIF4E, PABP and eIF4G [140,141]. VV appears to utilize SG components for different purposes. The G3BP1/Caprin1 complex aids VV transcription [140]; viral translation initiation is dependent on eIF4E/eIF4G/PABP; and the viral protein I3 associates with eIF4G to recruit viral ssDNA [142], suggesting that SG components may link VV transcription and translation [141]. TIA-1 is not sequestered in DNA factories [143]; however, infection with a VV mutant lacking E3L, which activates PKR, induces aggregates that contain TIA-1, eIF3b, G3BP1 and USP10, called antiviral granules (AVGs), given that they restrict viral replication [94].

### 3.2. Double-Stranded RNA (dsRNA) Viruses

The family *Reoviridae* is composed of non-enveloped virions with a 9–12 dsRNA segment genome. The prototypical member of the family, rotavirus, causes the shut off of host protein synthesis [144], and despite inducing eIF2α phosphorylation, SG assembly is not induced, likely due to the translocation of PABP from the cytoplasm to the nucleus [144]. The persistent phosphorylation of eIF2α during rotavirus infection is PKR-dependent as a consequence of the high amount of viral dsRNA in the cytoplasm [145]. By contrast, mammalian orthoreovirus (MRV) induces SG assembly during the early stages of infection, at a step between viral uncoating and viral mRNA transcription, and requires phosphorylation of eIF2α, which is important to promote virus replication [146]. However, despite high levels of phosphorylated eIF2α, SGs are disrupted at later times during MRV infection [147]. Recently, Carroll and colleagues showed that the nonstructural protein μNS is recruited to SGs, but its expression alone was not able to modulate assembly, suggesting a relationship between viral factories and SGs [148].

### 3.3. Positive-Sense Single Strand RNA ((+)ssRNA) Viruses

All members of the *Picornaviridae* family are composed of non-enveloped particles. Evidence indicates that poliovirus (PV) proteinase 2A induces assembly of SGs early post-infection (between 2 and 4 h) [16,149], which are dispersed later in the infection through the cleavage of G3BP1 by the PV 3C proteinase (3Cpro) [150]. Interestingly, at a later time post-infection, aggregates containing viral RNA and TIA-1, but excluding the bona fide SG components eIF4G and PABP, are observed, suggesting that TIA-1 aggregation is unlinked from SG formation [151,152]. Encephalomyocarditis virus (EMCV) and Coxsackievirus B3 (CVB3) also disrupt SGs by cleavage of G3BP1 through a mechanism similar to that used by PV [50,153]. By contrast, Theiler murine encephalomyelitis virus (TMEV) and mengovirus, a strain of EMCV, inhibit SG assembly through the expression of the leader (L) protein, maintaining the G3BP-1 intact [154,155]. A mutant mengovirus, in which the Zn-finger domain of L is disrupted, induces G3BP1 aggregation in a PKR-dependent manner [155], suggesting that a G3BP1-Caprin1-PKR complex could induce innate immune activation during mengovirus infection [156].

In 2005, McInerney and colleagues showed that Semliki Forest virus (SFV), a member of the *Togaviridae* family composed of enveloped virions, is able to induce eIF2α phosphorylation promoting SG assembly at early stages of infection [157]. However, at late times post-infection, concomitant to an increase in vRNA levels, SFV is no longer able to promote SG assembly [157]. It was determined that the SFV-induced SG disassembly is caused by the C-terminal domain of the viral nonstructural protein 3 (nsP3), which forms a complex with G3BP1, sequestering it into the vRNA replication complex [158]. Chikungunya virus (CHIKV) nsP3 also sequesters G3BP1 and forms specific virus-induced cytoplasmic foci that lack the SG marker eIF3 and are resistant to cycloheximide treatment [159]. Later, Scholte and colleagues demonstrated that G3BP2, a close relative of G3BP1 containing a similar domain architecture, which is recruited into SGs [160], colocalized with CHIKV nsP2/3 [161]. G3BP2-containing complexes differs from the replication and transcription complexes (RTCs), suggesting a role of G3BPs in the early step of the CHIKV replication cycle [161]. In contrast, Rubella virus (RUBV) induces G3BP1 aggregates that colocalize with viral ssRNA and non-structural viral protein P150, suggesting a potential post-replicative role in encapsidation [162]. Finally, the infection with Sindbis virus (SINV), another alphavirus, induces the assembly of G3BP1 aggregates, which interact with nsP2, nsP3 [163,164] and nsP4 [165]. In addition, it has been shown that SINV-derived vectors induce the assembly of bona fide SGs, containing TIA-1, eIF4E and eIF4G in a PKR-dependent manner [166].

The *Flaviviridae* family are composed of enveloped virions and West Nile virus (WNV) was the first virus reported to block SG assembly. Li and colleagues showed that the 3` stem loop in the viral genome is able to capture TIA-1 and TIAR [167]. In addition, Emara and colleagues expanded these observations to dengue virus DENV-infected cells, where TIA-1/TIAR are found in RTCs [168]. However, the chimeric WNV W956IC, which produces high levels of early viral RNA, activates PKR and subsequently induces the SG assembly [169]. In another report, Xia and colleagues showed that DENV-infected A549 cells were able to induce G3BP1 aggregates independently of TIA-1 [170]. In addition, a proteomic analysis found that G3BP1, G3BP2, Caprin1 and USP10 interact with DENV RNA [171], and it was reported that G3BP1, G3BP2 and Caprin1 regulate the translation of ISG mRNAs, thus protecting DENV replication from IFN-mediated antiviral effects [172]. Consistent with these findings, TIA-1 and TIAR were also recruited to sites of tick-borne encephalitis virus (TBEV) replication. Indeed, eIF2α becomes phosphorylated, and SGs containing G3BP1, eIF3 and eIF4B are induced in TBEV-infected cells [173]. On the other hand, the Japanese encephalitis virus (JEV) core protein was shown to sequester G3BP1 and USP10 through an interaction with Caprin1, resulting in the suppression of SG formation [174]. There is also evidence showing that infection with bovine viral diarrhea virus (BVDV), a *Flaviviridae* pestivirus, impairs the arsenite-mediated SG assembly; despite the fact that BVDV N-terminal protease (Npro) is able to interact with several RNA granules components, such as YB-1, IGFBP2, DDX3, ILF2 and RHA (DXH9) [175]. Contrasting the aforementioned flavivirus, HCV modulates the SG assembly by controlling the phosphorylation of eIF2α. Ariumi and colleagues reported evidence indicating that HCV infection delocalizes components of SGs (G3BP1, ATX2, PABP1) to lipid droplets (LDs), while Garaigorta and colleagues reported that HCV induces the assembly of bona fide SGs in a PKR-dependent manner. This report also demonstrated that TIA-1, TIAR and G3BP1 play a pivotal role in several steps of the HCV life cycle [176]. In addition, Ruggieri and colleagues observed rapid cycles of SG assembly and disassembly during the HCV infection depending on the eIF2α phosphorylation state [177]. They reported that while SG assembly is regulated by dsRNA promoting phosphorylation of eIF2α mediated by PKR, SG disassembly is regulated by a rapid eIF2α dephosphorylation through both protein phosphatase 1 (PP1) and its regulatory subunit, growth arrest and DNA-damage-inducible 34 (GADD34) [177]. Recently, another component of SGs, DDX3, has been reported to bind the HCV 3′ UTR and IKKα, leading to its activation to induce LD biogenesis [178]. However, late in infection, DDX3 and G3BP1 localize with the HCV core protein around LDs in order to initiate viral particle assembly [179,180]. These observations could explain the oscillation of SG assembly/disassembly detected in HCV-infected cells [177] and how SG formation is necessary for HCV RNA replication, assembly and egress [176,181,182].

The infection with Cricket paralysis virus (CrPV), a member of *Dicistroviridae* family, prevents the aggregation of Rox8 and Rin, Drosophila SG marker homologs of TIA-1 and G3BP-1, respectively, even in the presence of various stressor, such as arsenite, pateamine A or heat shock [183]. Although the CrPV 3C proteinase is sequestered in SGs, cleavage of the Rox8 or Rin is not detected in CrPV-infected cells [183]

The family *Coronaviridae* is composed of membrane-enveloped virions and one of the most significant features of coronaviruses is the expression of downstream genes via transcription of multiple 3` nested subgenomic mRNAs [184]. It has been shown that both mouse hepatitis coronavirus (MHV) and transmissible gastroenteritis virus (TGEV) induce TIA-1/TIAR aggregates with a concomitant increase in eIF2α phosphorylation levels [185,186]. MHV promotes SG assembly at early times post-infection [185], while TGEV induces SG assembly at later times post-infection [186]. During TGEV infection, the polypyrimidine tract-binding protein (PTB) is redistributed to the cytoplasm, associated with both TGEV gRNA and subgenomic mRNA (sgmRNA) and confined to TIA-1/TIAR aggregates [186].

Finally, *Caliciviridae* family is composed of non-enveloped virions and recently, Humoud and colleagues showed that the feline calicivirus (FCV) infection triggers eIF2α phosphorylation without inducing SG assembly [187]. This report demonstrated that the FCV viral 3C-like proteinase NS6 inhibit SG assembly by cleaving G3BP1 [187]. On the other hand, the murine norovirus 1 (MNV1) infection triggers eIF4E phosphorylation to control the translational machinery of the host cell [188], but does not impair SG assembly [187]

### 3.4. Negative-Sesnse Single Strand RNA ((-)ssRNA) Viruses

The family of *Orthomyxoviridae* is composed of enveloped virions with a segmented, negative ssRNA genome [184]. Influenza A virus (IAV) suppresses the assembly of SG during viral infection by expressing the non-structural protein NS1, which inhibits PKR activity [189]. The ability of IAV to interfere with SG assembly is reverted with the expression of an NS1-mutatant virus unable to bind dsRNA [189] or by expressing an NS1-deficient IAV, which promote SG assembly containing retinoic acid inducible gene I (RIG-I)-like receptors (RLRs), such as RIG-I, MDA5 and LGP2, or ISGs, such as PKR, RnaseL and OAS [39]. The NS1-mediated inhibition of SG assembly is dependent on the interaction with the RNA-associated protein 55 (RAP55) [190]. Besides NS1, the nucleoprotein (NP) and polymerase acidic protein X (PA-X) also contribute to block the SG assembly independent of eIF2α phosphorylation [191]. In addition, given that DDX3 has been implicated in the sensing of viral RNA to modulate IFN production and SG assembly (reviewed in [180]), Thulasi Raman and colleagues showed that DDX3 interacts with NP and colocalizes in SGs upon infection with an IAV mutant lacking NS1, suggesting a novel antiviral function for DDX3 [192].

Junin virus (JUNV) is a virus belonging to *Arenaviridae*, a family of enveloped virions with a bi-segmented negative-sense single strand RNA ((-)ssRNA) genome, that prevents SG assembly by impairing the phosphorylation of eIF2α [193], while the expression of JUNV nucleoprotein (N) and/or the glycoprotein precursor (GPC) are enough to inhibit SG assembly [193]. In addition, arenavirus RTCs contain ribosomal proteins L10a and S6 and translation initiation factors eIF4G and eIF4A and G3BP1 [194], suggesting a role of the SG component in the viral replication cycle.

Vesicular stomatitis virus (VSV), a virus in the family *Rhabdoviridae*, induces the phosphorylation of eIF2α and promotes the assembly of SG-like particles that contain TIA-1, TIAR, PCBP2, viral replication proteins and RNA, but not the eukaryotic initiation factor 3 (eIF3), nor eIF4A [195], suggesting that the SG-like structures may play a role in the VSV infection cycle.

Members of *Paramyxoviridae* family are composed of enveloped virions. Respiratory syncytial virus (RSV) induces SG assembly mediated by PKR, and its formation enhanced RSV replication [196,197]. Given that RSV infection forms cytoplasmic inclusion bodies (IBs) to mediate viral replication, Lindquist and colleagues observed that HuR, another SG component, localized to IBs [196] as well as MDA5 and MAVS proteins, suggesting a role in the suppression of IFN production [198]. Moreover, it was recently reported that RSV sequesters phosphorylated p38 (p38 P) and O-linked N-acetylglucosamine (OGN) transferase (OGT) into viral IBs, inhibiting the MAPK-activated protein kinase 2 (MK2) pathway and suppressing the assembly of SGs, respectively [199]. Measles virus (MeV) has the capacity to affect the innate and adaptive immune response through the accessory viral proteins V and C [200]. Interestingly, while infection with a protein C-deficient MeV efficiently induces SG assembly mediated by PKR, wild-type MeV does not [38]. However, wild-type MeV induces the assembly of SGs in ADAR1-knockdown cells, suggesting a role between the viral C protein and ADAR1 in modulating the assembly of SGs [38]. Sendai virus (SeV) infection slightly induces SG assembly, while short transcripts generated from the 3′-ends of antigenome RNA, SeV trailer RNA, interact with TIAR to prevent the assembly of SG [201]. In addition, recent data showed that SeV viral C protein also play a role in impairing SG assembly and IFN production [202]. This study revealed that SeV C-deficient recombinant 4C(-) infection forms SG-like structures containing RIG-I and unusual viral RNA species, suggesting that these structures could be reminiscent of anti-viral stress granules (avSGs) (reviewed in [180])

*Bunyaviridae* are a large family composed of enveloped virions with a tri-segmented (-)ssRNA genome [184]. The peculiarity of Bunyaviruses is that they require a capped oligonucleotide, which is scavenged from host mRNAs, to initiate its own mRNA synthesis, a process called “cap-snatching” [184]. These mechanisms take place in PBs, where the viral N protein binds to cap structures of cellular mRNAs, and the endonuclease domain of the RNA-dependent RNA polymerase (RdRp) cleaves cap-downstream sequences in a range of 10–18 ribonucleotides [203,204]. This capped-RNA fragment is used as a primer for mRNA synthesis by the viral RdRp. Interestingly, one report with Sin Nombre virus (SNV), a member of the hantaviruses genera, showed that the cellular mRNAs target of cap-snatching are mRNAs with a premature stop codon (PTC), which have been addressed to PBs for degradation through the non-sense mediated decay pathway (NMD) [205]. Another report with the Rift Valley fever virus (RVFV) from the Phlebovirus genera showed attenuation of the Akt/mTOR signaling pathway, which in turn increases the activity of 4EBP1/2 proteins leading to the arrest of cap-dependent translation [206]. Although SG formation would be expected upon inhibition of translation, this report demonstrated that SGs are instead disassembled during RVFV infection. It is noteworthy that during attenuation of Akt/mTOR 5′ TOP-containing mRNAs (including those coding for translation initiation factors), these are selectively targeted to SGs. Notably, the authors observed that 5′-end sequences from TOP mRNAs are included in RVFV’s mRNAs captured by the cap-snatching mechanism in PBs. Those data suggest that RVFV temporally induces SGs to nucleate important mRNAs and in turn promote mRNAs cargo from SGs to PBs where, finally, RVFV uses these cellular mRNAs for cap-snatching. Indeed, decay of the core of translation machinery would not represent a problem for bunyaviruses, since it has been described that SNV N protein is capable of replacing the entire eIF4F complex (eIF4E, eIF4A and eIF4G) and binding the 40S ribosomal subunit, thus recruiting the translation machinery to its own mRNAs [207,208]. Moreover, it is widely accepted that Bunyaviruses do not have a poly(A) tail at the 3′-end of their mRNAs. Indeed, there is evidence showing that Bunyamwera virus (BUNV) and Andes virus (ANDV) have a 3′ UTR, which replaces the poly(A) tail function, and interestingly, the translations of those mRNAs are poly(A) binding protein (PABP)-independent [209,210]. These findings show that bunyaviruses use complex mechanisms of transcription/translation that are potentially interconnected with cytoplasmic RNA granules.

On the other hand, Bunyaviral proteins, like other viral proteins, have evolved mechanisms to inactivate PKR and inhibit the IFN response. Similarly to the non-structural protein NS1 from IAV [211] or NS4A from Dengue virus [212], studies with Orthobunyaviruses, Hantaviruses and Phleboviruses have shown that the non-structural protein from the S segment (NSs) acts as an inhibitor of the IFN response [213,214,215,216], as well as glycoprotein Gn and the capsid N protein from Hantaviruses [217,218,219]. For example, the capsid N protein from Andes Hantavirus (ANDV) is an inhibitor of PKR dimerization, impairing its activation; however, the translation shut-off is not observed in Hantavirus-infected cells. [220]. In contrast, RVFV infection promotes a shut-off of global translation, where NSs protein mediate PKR degradation by the proteasome [221,222]. Undoubtedly, the field of cytoplasmic RNA granules related to the transcription and translation of bunyaviruses already began to show interesting findings, which will help to answer some ancient questions linked to the molecular mechanisms of its replication cycle, as it has been thought that transcription and translation are coupled like in prokaryotic systems [223,224]. However, the SG formation has not been addressed to date.

Recently, Nelson and colleagues showed that the infection with Ebola virus(EBOV), member of *Filoviridae* family composed of enveloped virions, does not trigger eIF2α phosphorylation or SG formation. However, SG components are sequestered within viral inclusions where they colocalize with viral mRNA [225]. In addition, viral protein VP35 not only prevents SG formation by blocking PKR activation, but also disrupts SG formation independently of eIF2α [225].

### 3.5. Single Strand RNA Retroviruses (ssRNA-RTs)

Retroviruses are enveloped, positive ssRNA viruses, which synthesize complementary DNA (cDNA) by reverse transcription that is integrated into the host chromosomal DNA [184]. The oncoretrovirus, human T-cell leukemia virus (HTLV-1), causes a blockade of SG assembly mediated by the viral regulatory protein, Tax. Legros and colleagues observed that Tax interacts with histone deacetylase 6 (HDAC6), a critical component of SG, to block SG assembly [226]. Tax also interacts with USP10, inhibiting SG assembly and enhancing the production of reactive oxygen species [227]. On the other hand, human immunodeficiency virus type 1 (HIV-1) significantly impairs SG assembly in favor of the assembly of Staufen1-containing HIV-1-dependent ribonucleoproteins [228]. Indeed, we showed that HIV-1 Gag blocks SG assembly irrespective of eIF2α phosphorylation [229]. In addition, we reported that the interaction between the N-terminal domain (NTD) of the capsid domain of Gag (p24) and host eukaryotic elongation factor 2 (eEF2) are critical for the SG blockade, while that eEF2 depletion not only lifted the SG blockade, but also resulted in impaired virus production and infectivity [229]. Interestingly, we also reported that HIV-1 Gag mediates the disassembly of preexisting SGs via an interaction with G3BP1 [229], and more recently, it was shown that G3BP1 binds HIV-1 unspliced mRNA (gRNA) in the cytoplasm of macrophages to inhibit viral replication [230]. At the same time, we reported that the HIV-1 unspliced mRNA (gRNA) promotes the assembly of a pre-translation initiation intermediate with DDX3, another SG component, and a subset of translation initiation factors, such as eIF4GI and PABPC1, suggesting that these intermediates may serve to concentrate the gRNA and eIFs in order to enhance the efficiency of polysome association [231]. It is noteworthy that the mechanism associated with SGs-blockage by HIV-1 Gag was dependent on the kind of stressor. As such, sodium selenite (Se) causes 4EBP1-mediated mRNA translational arrest and the subsequent assembly of non-canonical type II SGs [232]. Recently, Cinti and colleagues showed that in HIV-1-expressing cells under Se treatment, Gag interacts with eIF4E, reducing hypophosphorylated 4EBP1 associated with the 5′ cap in order to promote the disassembly of SGs [233]. In contrast to HIV-1, the replication of human immunodeficiency virus type 2 (HIV-2) induces the spontaneous assembly of SG [231]. As such, the HIV-2 gRNA recruits TIAR to form a novel viral mRNP, where the switch between translation to packaging could occur [231].

## 4. Viral Infections and Processing Bodies

Several viruses are known to disrupt P-bodies. Here, we will summarize the most up-to-date information known about the relationships between viruses and PBs (Table 3). 

### 4.1. dsDNA Viruses

Adenovirus regulates its gene expression over the time of infection, triggering an accumulation of viral late mRNA in the cytoplasm [184]. To prevent viral mRNA degradation, the early protein E4 11k binds Rck/p54/DDX6, relocalizing it with PB components such as Lsm-1, Ge-1, Ago2 and Xrn1, to aggresomes, sites where these proteins are inactivated [237]. In contrast, within the human papilloma virus (HPV), the oncoprotein E6 commands the re-colocalization of PKR in PBs, suggesting an antiviral effect for these granules [46]. Finally, during HCMV infection, PB components, such as Dcp1a, Edc4, Rck/p54/DDX6 and RAP55, increased in a translation-independent manner requiring cellular, but not viral RNA synthesis [238]. 

### 4.2. dsRNA Viruses

A recent report showed that rotavirus infection triggers a significant time-dependent decline of PBs components XRN1, DCP1, Pan3, but not GW182 [239]. Pan3, but not XRN1 and DCP1, undergoes accelerated turn over in response to rotavirus infection, while XRN1 and DCP1 were translocated to the nucleus [239], as well as PABP [144].

### 4.3. (+)ssRNA Viruses

In the case of flaviviruses, WNV and DENV infections reveal a reduction in PB formation [168]. Indeed, WNV sequesters several components of PBs, such as Lsm1, GW182, Xrn1, DDX3 and Rck/p54/DDX6, in viral replication factories to promote viral replication [240]. Furthermore, it has been shown that Rck/p54/DDX6 binds a stem-loop present in the DENV 3′ UTR, inhibiting PB formation [171]. Interestingly, flaviviruses (including yellow fever virus (YFV) and DENV) generate a subgenomic flavivirus RNA (sfRNA), as a product of gRNA degradation by Xrn1 at pseudoknot 3 [241]. The sfRNA and Xrn1 colocalized in PBs and were essential for viral cytopathogenicity [242]. In addition, Moon and colleagues showed that inhibition of XRN1 by sfRNA-interaction results in accumulation of uncapped cellular mRNA [243]. Likewise, HCV infection relocalized PB components to viral factories close to lipid droplets, such as RCK/p54/DDX6, Lsm1, Xrn1, PatL1, Ago2 and DDX3 [181,182]. The depletion of these components has a detrimental role over HCV replication [181,182,244], while RCK/p54/DDX6, Lsm1 and PatL1 play a central role in HCV translation and replication [245,246]. However, the disruption of PB mediated by depletion of Rap55 does not affect HCV replication [247]. Together, these data strongly suggest that HCV co-opts several PB components to ensure viral replication, but if PBs are formed, they do not have an inhibitory effect during viral infection.

On the other hand, infection with picornaviruses, such as PV and CVB3, entirely disrupts PB formation through the virally-induced cleavage of Dcp1a mediated by viral protease 3C, as well as the degradation of Xrn1 and Pan3 mediated by the proteasome [248]. It has been recently shown that expression of PV-protease 2A also blocks PB formation more efficiently than protease 3C [149]; however, the mechanism remains unclear. Furthermore, CrPV infection only disrupts GW182/Dcp1 aggregates, but not Ago1/Ago2, suggesting that these PBs components could have a differential role in viral infection [183]. Moreover, the U-rich region close to the 3′-end of SINV mRNAs interacts with HuR, generating a dramatic translocation of the host protein out of the nucleus, stabilizing viral transcripts during infection and subsequently preventing the assembly of PBs [249].

### 4.4. (-)ssRNA Viruses

The negative-strand RNA viruses are known to use cap-snatching mechanism, to initiate its own mRNA synthesis. However, for IAV, this process occurs in the nucleus, while that for Bunyavirus occurs in PBs. Mir and colleagues showed that hantavirus nucleocapsid protein (N) avoids the 5′ cap degradation of cellular mRNAs, protecting them from Dcp1a/Dcp2-mediated decapping, [204]. In addition, the interaction of N protein with 5′-Cap, instead of eIF4E/eIF4F, allows the Hantavirus transcripts to escape from PB and recruit ribosomes [207]. In the case of IAV, the interaction of RAP55 with NS1 impairs PB formation, but also prevents the capture of NP in the PBs [190].

### 4.5. ssRNA-RT Viruses

Lastly, the protein APOBEC3 (apolipoprotein B mRNA editing enzyme catalytic polypeptide-like 3) provides anti-HIV-1 activity while being a PB component that interacts with Ago2, Dcp1a, Dcp2 and DDX6 [47,115]. However, the viral infectivity factor Vif protein induces APOBEC3 degradation, preventing its incorporation into virions [47], affecting its subcellular localization, degradation rates and antiviral properties [250]. Several groups have reported that depletion of PB components or RISC components (including DDX6, LSM-1, GW182, XRN1, DGCR8, Dicer and Drosha) increases the viral production and that gRNA and Gag protein localize to PBs [251,252,253]. Nevertheless, gRNA localization in PBs has not been detected by others [228,254], and the depletion of Ago2 or DDX6 resulted in the inhibition of HIV-1 replication [255,256]. Likewise, Abrahamyan and colleagues reported a dramatic decrease in the abundance of PBs around gRNA-foci in HIV-1-expressing cells, even in cells treated with sodium arsenite [228], suggesting a relocalization of PB during the HIV-1 infection. Given that the events of HIV-1 capsid assembly have not been associated with PBs, Reed and colleagues demonstrated that assembly intermediates (AIs), containing HIV-1 Gag, GagPol and Vif [257], are formed by the recruitment of DDX6 and ATP-binding cassette protein E1, ABCE1 [256]. On the other hand, the overexpression of Mov10, a putative RNA helicase that associates with APOBEC in RISC complexes and that was found in PBs, can inhibit viral replication and reduce Gag expression [258,259]. In addition, it was reported that the recruitment of Mov10 and APOBEC3G into virions is independent of localization on their PBs [260]. However, given the discrepancy between these reports, more work is needed to elucidate the role of PBs and their components during the HIV-1 replicative cycle.

## 5. Conclusions

Although significant advances have been made to understand how viruses modulate the assembly/disassembly of RNA granules, there are still outstanding questions that need to be addressed. For example, could these mechanisms be targets of new antiviral drugs? What are the molecular mechanisms and/or signaling pathways that transport mRNAs from one RNA granule to another? Do post-translational modifications that serve as signals to modulate the formation of RNA granules exist? To answer these questions, several reports have helped us to comprehend the molecular biology of RNA granules [261,262,263,264,265,266,267,268,269,270], however, further work is necessary to determine the viral mechanisms that modulate the RNA granules. In addition, emerging evidence has related RNA granules with innate antiviral immunity as part of the integrated stress response. Finally, understanding how viruses counter anti-viral stress responses lays the groundwork for new strategies to bolster host cell immune defenses against invading pathogens.

## Figures and Tables

**Table 1:** Components of stress granules (SGs) and processing bodies (PBs).

**SG Component**	**Reference**	**SG Component**	**Reference**
ADAR	[38,50]	MEX67	[51]
AKAP350	[52]	MLN51	[53]
ANG	[54]	MSI1	[55]
ATXN2/pbp1	[56,57]	mTOR	[37,58]
CALR	[59]	OGFOD1	[60]
Caprin1	[61]	P97/NAT1	[62]
CCAR1	[52]	PABP1	[30]
CIRP	[63]	PHB2	[64]
CUGBP1	[65]	PKP1/3	[66]
DDX1	[67]	PKR	[38,50]
DDX3/Ded1	[51]	PMR1	[33]
DHC1	[68]	PRTB	[69]
DISC1	[70]	PUM1	[71]
eIF2B	[72]	PUM2	[73]
eIF2α	[74]	RACK1	[75]
eIF3	[17]	RBM42	[76]
eIF4A1	[17]	RHAU/DHX36	[36]
eIF4G	[17]	RIG-I	[38,50]
eIF5A	[77]	RNH1	[54]
FAK	[78]	Rpp20	[79]
FBP/KSRP	[80]	RSK-2	[81]
FUS	[82]	Sam68	[83]
FXR1/FXMR	[31]	SERBP1	[84]
FXR2P	[31]	SGNP	[85]
G3BP1	[20]	SMN	[34]
G3BP2	[86]	Staufen1	[35]
Grb7	[78]	TDP43	[87]
HDAC6	[88]	TDRD3	[84]
hnRNP A1	[89]	TFE3	[90]
hnRNP K	[76]	TFEB	[90]
Hsp27	[91]	TRAF2	[92]
HuD	[93]	USP10	[21,94]
HuR	[95]	Vinexin	[96]
IFIH1/ MDA-5	[38,50]	ZBP-1	[97]
IP5K	[98]		
KHC/KLC	[68]		
LINE1 ORF1p	[99]		
MBNL1	[67]		
**PB Component**	**Reference**	**PB Component**	**Reference**
Ccr4	[100]	Htt	[101]
Dcp1/Dcp2	[100]	LSM1	[40]
Ebs1	[102]	Pan2/3	[103]
Edc1-2	[104]	Pat1/PatL1	[100]
Edc3	[105]	Pop2/Caf1	[106]
eIF4E-T	[107]	PRMT1	[108]
Ge-1/Hedls	[41]	TNRC6B	[109]
GW182	[110]	UPF1	[42]
hMex3A	[111]	UPF2	[42]
hnRNP A3	[112]	UPF3	[42]
**SG and PB Component**	**Reference**	**SG and PB Component**	**Reference**
Ago1	[48]	NXF7	[112]
Ago2	[113]	PABP/Pab1	[30,114]
APOBEC3G	[47,115]	PCBP2	[116]
BRF1	[117]	Rap55/Scd6	[118]
CPEB	[32]	RCK/Dhh1/DDX6	[32]
Dcp1/Dcp1a	[32]	Roquin	[119]
eIF4E	[17]	Smaug 1	[120,121]
FAST	[117]	TIA1/TIAR	[30,91,122]
hMex3B	[123]	TTP/BRF1	[117]
hnRNP Q	[124]	Xrn1	[117]
IPO8	[125]	YB-1	[126]
JNK	[127]		
Lin28	[128]		

**Table 2:** Virus families that modulate SGs.

	Genome	Virus Family	Virus	SG Induction	SG Blockade	Mechanism	Reference
I	dsDNA	*Herpesviridae*	Herpes simplex virus type 1 (HSV-1)	No	Yes	(-)RNA stem loop captures TIA-1/TIAR to favor replication	[133]
			Herpes simplex virus type 2 (HSV-2)	Yes	Yes	Inhibits SG assembly dependent of eIF2α-P	[136]
						Induces SG independent of eIF2α-P	[136]
			Cytomegalovirus (HCMV)	No	Yes	Induces UPR with intact viral translation	[138]
		*Poxviridae*	Vaccinia virus (VV)	Yes	Yes	Replication factories (RF) sequester G3BP1, Caprin1	[140]
						RF sequester eIF4G, eIF4E, PABP	[141]
						VV lacking of E3L induces antiviral granules (AVGs)	[94]
III	dsRNA	*Reoviridae*	Rotavirus	No	Yes	NSP2, VP2 and NSP5 translocate PABP to the nucleus	[144]
			Mammalian orthoreovirus (MRV)	Yes	Yes	Induces eIF2α-P	[234]
						uNS is recruited to SGs	[147,148]
IV	(+)ssRNA	*Picornaviridae*	Poliovirus (PV)	Yes	Yes	Early PV-infection induces SG assembly	[16]
						viral C3 protease cleaves G3BP1	[150]
						PV-infection induces TIA-1 aggregates	[151]
			Encephalomyocarditis virus (EMCV)	No	Yes	Cleavage of G3BP1	[50]
			Coxsackievirus B3 (CVB3)	No	Yes	Cleavage of G3BP1	[153]
			Theiler’s murine encephalomyelitis virus (TMEV)	No	Yes	Leader protein (L) inhibits SG assembly	[154]
			Mengovirus, a strain of EMCV	No	Yes	Leader protein (L) inhibits SG assembly	[155,156]
		*Togaviridae*	Semliki Forest virus (SFV)	Yes	Yes	Induces eIF2α-P	[157]
						nsP3 protein captures G3BP1	[158]
			Chikungunya virus (CHIKV)	No	Yes	nsp3 protein recruits G3BP1 to replication foci	[159]
						G3BP2 colocalize with nsP3/nsP2	[161]
			Rubella virus (RUBV)	Yes	No	Accumulation of G3BP	[162]
			Sindbis virus (SINV)	Yes	Yes	Nsp4 interacts with G3BP1	[165]
						Induces PKR-mediated SG assembly	[166]
		*Flaviviridae*	West Nile Virus (WNV)	No	Yes	3′-end viral genome captures TIA-1/TIAR	[167]
			Dengue virus (DENV)	No	Yes	3′-end viral genome captures TIA-1/TIAR	[168]
						3′ UTR interacts with G3BP1, G3BP2, Caprin1 and USP1	[171]
			Tick-borne encephalitis virus (TBEV)	Yes	No	Induces eIF2α-P	[173]
			Japanese encephalitis virus (JEV)	No	Yes	Core protein interacts with Caprin1	[174]
			Bovine viral diarrhea virus (BVDV)	No	Yes	Impairs the Ars-mediated SG assembly	[175]
			Hepatitis C virus (HCV)	Yes	Yes	G3BP1, ataxin-2 and PABP localized to lipid droplets	[181]
						Induces PKR	[176]
						SG disassembly mediated by GADD34	[177]
						DDX3 binds 3′ UTR	[178]
						DDX3 and G3BP1 localize with HCV core protein	[179]
		*Dicistroviridae*	Cricket paralysis virus (CrPV)	No	Yes	3Cpro sequesters to SG	[183]
		*Coronaviridae*	Mouse hepatitis coronavirus (MHV)	Yes	No	Induces eIF2α-P	[185]
			Transmissible gastroenteritis virus (TGEV)	Yes	No	PTB localizes to SG and correlates with replication increase	[186]
		*Caliciviridae*	Murine Norovirus 1 (MNV1)	nd	No	eIF4E phosphorylation	[188]
			Feline Calicivirus (FCV)	No	Yes	Cleavage of G3BP1 by FCV NS6	[187]
V	(-)ssRNA	*Orthomyxoviridae*	Influenza A virus (FLUA)	No	Yes	NS1 inhibits PKR and eIF2α-P	[189,190]
						NP and PA-X block SGs	[191]
						DDX3 colocalize with NP	[192]
		*Arenaviridae*	Junin virus (JUNV)	No	Yes	N and GPC proteins impairs SG assembly	[193,194]}
		*Rhabdoviridae*	Vesicular stomatitis virus (VSV)	Yes	No	Induces SG-like structures recruiting TIA-1, TIAR y PCBP2	[195]
		*Paramyxoviridae*	Respiratory syncytial virus (RSV)	Yes	Yes	Induces PKR	[196,197]
						5′ trailer region induces eIF3-aggregates	[235]
						RSV sequesters p38-P and OGN	[199]
			Measles virus (MeV)	Yes	nd	Induces PKR	[38]
						viral C protein and ADAR1 modulate SGs	[38]
			Sendai virus (SV)	Yes	Yes	Trailer RNA captures TIAR from SGs	[201]
						Forms antiviral stress granules (avSG)	[202]
		*Bunyaviridae*	Rift Valley fever virus (RVFV)	Yes	Yes	attenuate Akt/mTOR signaling	[206]
			Andes hantavirus (ANDV)	nd	Yes	N protein inhibits PKR activation	[220]
		*Filoviridae*	Ebola virus	No	Yes	VP35 prevents SG formation by blocking PKR activation	[225]
VI	ssRNA-RT	*Retroviridae*	Human T-cell leukemia virus (HTLV-1)	No	Yes	Tax protein interacts with HDAC6	[226]
						Tax protein interacts with USP10	[227]
			Human immunodeficiency virus type 1 (HIV-1)	No	Yes	Staufen 1 and Gag block SG assembly	[228];
						EEF2 interacts with Gag to blocks SG assembly	[229]
						G3BP1 interacts with Gag to disassembly SG-preformed	[229]
						gRNA promote pre-translation initiation complex	[236]
						Gag interacts with eIF4E to promote disassembly of SGs	[233]
			Human immunodeficiency virus type 2(HIV-2)	Yes	No	gRNA and TIAR aggregates in SG	[231]

nd = not determined; dsDNA = double-stranded DNA; dsRNA = double-stranded RNA; UPR = unfolded protein response; (+)ssRNA = positive-sense single strand RNA; (-)ssRNA = negative-sense single stand RNA; ssRNA-RT = single strand RNA retroviruses

**Table 3:** Virus families that modulate PBs.

	Genome	Virus Family	Virus	PB Induction	PB Blockade	Mechanism	Reference
I	dsDNA	*Adenoviridae*	Adenovirus	No	Yes	Decreased PB by redistribution of E4 11K	[237]
		*Papillomaviridae*	Human papilloma virus (HPV)	Yes *	No	Re-colocalization of PKR in PBs	[46]
		*Herpesviridae*	Cytomegalovirus (HCMV)	Yes	No	Increased of Dcp1a, EDC4, Rck/p54/DDX6 and Rap55 protein levels	[238]
III	dsRNA	*Reoviridae*	Rotavirus	No	Yes	Decrease of XRN1, DCP1 and Pan3, but not GW182 protein levels	[239]
IV	(+)ssRNA	*Flaviviridae*	West Nile virus (WNV)	No	Yes	Captures of Lsm1, GW182, DDX6, DDX3 and Xrn1 to viral replication factories (RF)	[168,240]
			Dengue virus (DENV)	No	Yes	Captures of Lsm1, GW182, DDX6, DDX3 and Xrn1 to viral replication factories (RF)	[168,240]
			Yellow fever virus (YFV)	Yes *	No	sfRNA stalls Xrn1 and co-localizes at PB	[241]
			Kunjin virus (KUNV), Australian strain of DENV	Yes *	No	sfRNA stalls Xrn1 and co-localizes at PB	[242]
			Hepatitis C virus (HCV)	Yes *	Yes	DDX6, Lsm1, Xrn1, PATL1 and Ago2 localize to lipid droplets	[181]
						Dcp2 not localize to viral factories	[181]
		*Picornaviridae*	Poliovirus (PV)	No	Yes	Cleavage of Xrn1, Dcp1a and Pan3	[248]
						Protease 2A blocks PB formation	[149]
			Coxsackievirus B3 (CVB3)	No	Yes	Cleavage of Xrn1, Dcp1a and Pan3	[248]
		*Dicistroviridae*	Cricket paralysis virus (CrPV)	Yes	Yes	Disrupts only GW182/Dcp1 aggregate, but not Ago1/Ago2	[183]
		*Togaviridae*	Sindbis virus (SINV)	No	Yes	HuR-translocation out of the nucleus	[249]
V	(-)ssRNA	*Orthomyxoviridae*	Influenza virus A (IAV)	No	Yes	Interaction of RAP55 and NSP1	[190]
		*Bunyaviridae*	Hanta virus	Yes *	No	Cap snatching occurs in PBs	[204]
VI	ssRNA-RT	*Retroviridae*	Human immunodeficiency virus type 1 (HIV-1)	nd	Yes	HIV-1 mRNA interacts with DDX6, Ago 2 and APOBE3G and displaces from the PB	[253]
						Relocalization of PB during the HIV-1 infection	[228]
						Assembly intermediates (AIs) recruits DDX6 and ABCE1	[256]
						Overexpression of MOV10 inhibits HIV-1 replication	[258]

*maintains PB endogenously; nd = not determined; dsDNA = double-stranded DNA; dsRNA = double-stranded RNA; UPR = unfolded protein response; (+)ssRNA = positive-sense single strand RNA; (-)ssRNA = negative-sense single stand RNA; ssRNA-RT = single strand RNA retroviruses.
